# Parenting and mobile phone addiction tendency of Chinese adolescents: The roles of self-control and future time perspective

**DOI:** 10.3389/fpsyg.2022.985608

**Published:** 2022-10-12

**Authors:** Yuan Peng, Yali Wang, Shaozhuo Liu, Xingzhao Hu

**Affiliations:** School of Teaching, Xi'an University, Xi'an, China

**Keywords:** mobile phone addiction tendency, parenting, self-control, future time perspective, the moderated mediation model

## Abstract

Previous studies examined the impact of parenting on adolescents' mobile phone addiction tendencies. However, relatively few studies examined the potential mechanism underlying such a relationship. Thus, the present study further explored the mediation effect of self-control and the moderating effect of future time perspective between parenting and mobile phone addiction tendencies of Chinese adolescents. A sample of 1,349 Chinese adolescents (*M*_age_ = 15. 22 years, SD = 0.79) responded to the anonymous questionnaires regarding parenting, including parental control and parental care, self-control, future time perspective, and mobile phone addiction tendency. The results showed that (1) parental control was positively related to adolescents' mobile phone addiction tendencies, while parental care was negatively related to adolescents' mobile phone addiction tendencies; (2) self-control could mediate the pathway from both parental control and parental care to adolescents' mobile phone addiction tendencies; and (3) the indirect pathway could be moderated by future time perspective. Specifically, a high future time perspective combined with high self-control predicted a low level of mobile phone addiction tendency. In contrast, low self-control was associated with a high tendency toward mobile phone addiction, regardless of their future time perspective. The present study revealed a complex interplay between family and individual factors contributing to adolescents' mobile phone addiction tendencies.

## Introduction

Mobile phones have become indispensable worldwide due to their versatility and portability (Burchell, 2015). According to recent data, 99.6% of internet users in China would surf the internet through mobile phones by August 2021 (CNNIC, 2021). Moreover, the adoption of mobile phones has steadily increased worldwide. Although it provides great convenience, the excessive use of mobile phones may result in various problems such as mobile phone addiction tendency (Billieux, 2012; Badenes–Ribera et al., 2019; Volkmer and Lermer, 2019). Mobile phone addiction tendency, which is defined as excessive dependence and abuse of a mobile phone, is broadly viewed as a subset of behavioral or technological addiction (Billieux, 2012). Adolescents appear to be enthusiastic users of mobile technologies and are particularly at risk of developing mobile phone addiction (Liu et al., 2020; Sun et al., 2020). Numerous studies suggested that mobile phone addiction predicts a series of adverse outcomes, such as academic failure, impaired physical and mental health, and compromised social functioning (Echeburúa and Corral, 2010; Liu and Wang, 2011; Billieux, 2012; Lepp et al., 2014; Chen et al., 2016; Jiang and Zhao, 2016; Griffiths, 2017; Liu et al., 2020). Therefore, many efforts were made to clarify the influencing factors and potential mechanisms of adolescents' mobile phone addiction tendencies, which are conducive to formulating prevention and intervention strategies.

Empirical studies showed significant differences between mobile phone addiction and no mobile phone addiction in adolescents in individual and family environmental factors, but there are few studies on how these two factors jointly affect adolescents' mobile phone addiction tendencies. The present study explored how both family and individual factors contribute to adolescents' mobile phone addiction tendencies.

Based on the component model of behavioral addiction (Griffiths, 2005), environmental factors and individual factors jointly contribute to behavioral addiction, including mobile phone addiction (Bae, 2015). In addition, a family environment continues to shape and affect the behavior of adolescents. As the core variable of the family environment, parenting plays an important role in the psychosocial functioning of adolescents (Turner et al., 2009; Hou et al., 2020). Parenting or parental rearing behavior describes how parents use socialization practices to guide their children, such as how to respond to a child's needs and use discipline strategies to change the behavior of the child (Barber, 1996; Mcleod et al., 2007). Parental rearing behavior can be categorized into two broad classes: parental control and parental care (Markus et al., 2003). Parental control is characterized by over-protection, restriction, and rejection, which means that parents excessively interfere with or control the behavior of children and try to guide them on how to think and feel. In contrast, parental care is characterized by acceptance, identification, and emotional warmth (Markus et al., 2003; Mcleod et al., 2007). Many studies found that parental control was positively associated while parental care was negatively associated with adolescents' and young adults' mobile phone addiction (Mousavi et al., 2015; Lee et al., 2016; Lian et al., 2016). Besides the direct relationship between parental control/parental care and mobile phone addiction tendency, the mediation and moderation paths that underlie such association have gradually attracted much attention. The present study explored the possible mechanism between parental control/parental care and mobile phone addiction tendencies among Chinese adolescents.

### Self-control as a mediator

One of the most critical abilities of an individual is to regulate the present behavior to accomplish long-term goals, which involves self-control. Evidence shows that individuals with strong self-control are more successful in multiple fields such as academics, vacations, social situations, and health (Gottfredson and Hirschi, 1990; Muraven and Baumeister, 2000). In view of its essential role in guiding human behavior, the nature of self-control has attracted much attention (Muraven and Baumeister, 2000). Self-control is the ability to top-down adjust individual desires or behaviors to adapt to the environment (Tangney et al., 2004), including choosing appropriate behavior and inhibiting inappropriate behavior (Ent et al., 2015). Self-control could potentially mediate between parental rearing behavior and adolescents' mobile phone addiction tendencies. However, the lack of self-control is one of the critical aspects of addiction (Davis, 2001). Based on the self-control theory (Gottfredson and Hirschi, 1990), low self-control occurs due to the aim of individuals to seek immediate gratification. Low self-control is a primary factor for many problematic behaviors, including violence, taking a risk, and internet addiction (Slater, 2003; Özdemir et al., 2014), and individuals with poor self-control are sensitive to immediate gratification and rewards (Gottfredson and Hirschi, 1990; Niemz et al., 2005; Kim et al., 2008). Thus, they are more likely to indulge in tempting, immediately gratifying rewards, and impulses of the virtual world. As previous studies on internet addiction suggest, similar to mobile phone addiction, individuals who are addicted to the internet cannot practice self-control or self-regulate and cannot set long-term goals (Baumeister et al., 2010; Shek and Lu, 2012; Akn et al., 2015). Previous studies found that self-control related to impulsiveness predicts internet addiction or mobile phone addiction tendencies (Van Deursen et al., 2015). Individuals with poor self-control are more likely to engage in excessive mobile phone usage, thus leading to mobile phone addiction tendency. In contrast, high self-control could be a highly indispensable protection factor against stimuli and temptation from the virtual world (Servidio, 2019) and reduce the risk of mobile phone addiction (Jiang and Zhao, 2016).

Parents play an essential role in the development of adolescents, including self-control ability. The self-control theory (Gottfredson and Hirschi, 1990; Hirschi and Gottfredson, 1993) proposed that parenting plays a role in developing children and self-control in adolescents, and ineffective parenting is regarded as a social environment that hinders an individual's development of self-control. Moreover, Chinese traditional culture has always emphasized the ability to suppress impulses. In such a cultural context, Chinese parents tend to implement strict socialization discipline and are more likely to use excessive interference or ridicule to punish children, which hinders children's internalization of control ability (Xing et al., 2017). In contrast, parental care, which provides independent support and warmth, may help children successfully internalize the requirements and goals of their parents and then facilitate the development of self-control. Parental control, such as refusal and over-protection, was found to reduce children's self-control (Barber, 2002; Crosswhite and Kerpelman, 2012; Li et al., 2013; Cecil et al., 2018). Parental care, such as warmth, was found to facilitate self-control in children (Finkenauer et al., 2005; Li et al., 2014). Therefore, parental control may undermine the development of autonomy and self-control in Chinese adolescents. In comparison, perceived parental care may foster self-control ability in Chinese adolescents.

Given the association between parental control/parental care, self-control, and mobile phone addiction tendency, the present study posited the following hypothesis:

**Hypothesis 1**: Self-control could mediate the relationship between parenting behavior (including parental control and parental care) and Chinese adolescents' mobile phone addiction tendencies.

### Future time perspective as a moderator

Although parenting would be expected to affect adolescents' mobile phone addiction tendencies through the self-control of adolescents, there may be several potential moderators underlying such indirect connection. Future time perspective, which has been regarded as a trait-like characteristic, focuses on the tendency of an individual to think, anticipate, and construct the future and the general attitude toward the future (Andre et al., 2018; Kooij et al., 2018). Future time perspective is closely related to an individual's emotion, cognition, motivation, and social adaptation (Kooij et al., 2018).

Zimbardo and Boyd (1999) proposed that adolescence was seen as a period when future time perspective increases to meet the need for transitioning into adulthood (Romer et al., 2010). According to the expectation value theory (Pang et al., 2014), the behavior motivation of an individual is determined by result expectation and value evaluation. Future time perspective could encourage individuals to realize the effectiveness of the current behavior and its more valuable future results, so they can better adjust to the current behavior. Therefore, an individual with a high level of future time perspective could adjust current behaviors by keeping in mind future goals (Song, 2004; Joireman et al., 2012).

It is reasonable to expect that future time perspective would moderate the indirect path between parenting, self-control, and mobile phone addiction tendency. Based on the dual-motive model of self-control (Fujita, 2011), the relative incentive value of a proximal and a distal goal is essential for self-control. If the incentive value of the distal goal outweighs that of the proximal goal, then self-control will likely be successful (Dreves and Blackhart, 2019). A future time perspective was suggested to adjust the relative incentive value of competing for proximal and distal goals. Specifically, a high future time perspective may lead individuals to consider the value of long-term goals, which help them more likely to prioritize long-term goals and further facilitate self-control. Accordingly, high self-control combined with a high future time perspective would make individuals less likely to pursue the incentive value of short-term goals and immediate satisfaction such as mobile phone use. Moreover, according to the strength model of self-control (Baumeister et al., 2007), self-control is regarded as a finite resource, and the exhaustion of self-control resources may lead to maladjustment. However, researchers found that motivation can compensate for the exhaustion of self-control resources (Muraven, 1998; Muraven et al., 2006). If individuals whose self-control resources are consumed can improve their motivation level, they will perform better in self-control tasks (Muraven, 1998; Muraven et al., 2006). As an influential motivating factor, a high future time perspective may compensate for the exhaustion of self-control resources. That is, when the future time perspective level of an individual is high, the protective effect of self-control on mobile phone addiction may be strengthened. Thus, future time perspective may moderate the impact of self-control on mobile phone addiction tendency. The specific hypothesis is as follows:

**Hypothesis 2**: Future time perspective could moderate the indirect effect of self-control on the relationship between parenting and Chinese adolescents' mobile phone addiction tendencies.

### The present study

**Figure 1:**
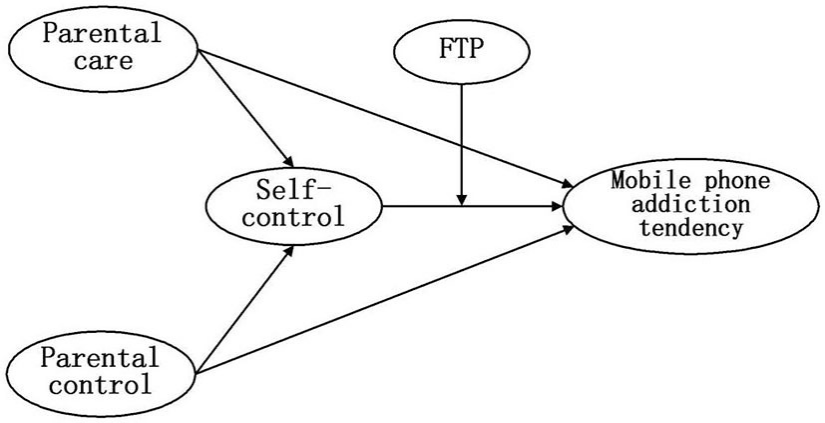
Proposed moderated mediation analysis of self-control on the relationship between parental control and parental care and adolescents' mobile phone addiction tendencies conditioned by future time perspective (FTP).

The present study first tested the mediation effect of self-control on the relationship between parental rearing behavior, including parental control and parental care and Chinese adolescents' mobile phone addiction tendencies. Second, it tested the moderation effect of future time perspective on the indirect pathway between parental rearing behavior and self-control and Chinese adolescents' mobile phone addiction tendencies. Together, these two research questions formed a moderated mediation model to examine how parental control and parental care contributed to mobile phone addiction tendency and when this association was most potent (see Figure 1).

## Method

### Participants

The Ethics Committee of Xi'an University approved the present research. Two welltrained postgraduates were present in each classroom (approximately 50 students) and explained the content, procedure, and confidentiality rules. Participants who took part in this survey voluntarily signed the informed consent form. After obtaining informed consent from adolescents, a questionnaire survey was conducted. A total of 1,360 students (excluding 11 invalid samples) were recruited from two middle schools in a city in North West China. Finally, 1,349 participants filled out the survey (*M*_age_ = 15, 22 years, SD = 0.79, and range = 13–16 years). In total, 56.30% of the participants were girls. All the participants were from the ethnic group Han and spoke Mandarin Chinese. The participants reported the household income using a 4-point scale (1 = <¥3,000, 2 = ¥3,000–¥7,000, 3 = ¥7,000–¥10,000, and 4 = more than ¥10,000) and the education level of parents on a 7-point scale (1 = lower than elementary school, 2 = elementary school, 3 = junior high school, 4 = high school, 5 = college or university, 6 = master's degree, and 7 = doctoral degree). An index of socioeconomic status (SES) of the participants was calculated consistent with previous studies (Cohen et al., 2006): (a) the father's education level (*M* = 3.57, SD = 1.09), (b) the mother's education level (*M* = 3.36, SD = 1.17), and (c) household income (*M* = 2.12, SD = 1.06). Survey completion took approximately 20 min. These participants were not clinical samples.

### Measures

#### Mobile phone addiction tendency

Mobile phone addiction tendency was assessed by the Mobile Phone Addiction Index (MPAI; Leung, 2008). This scale has four dimensions: inability to control cravings (7 items, e.g., “I tried to spend less time on the phone but failed.”), anxiety and feeling lost (5 items, e.g., “If I do not have a mobile phone, I'm worried that my friends or families will not be able to contact me. ”), withdrawal and escape (3 items, e.g., “When I feel lonely, I use my mobile phone to chat with others.”), and productivity loss (2 items, e.g., “I find myself obsessed with my mobile phone when I have other things to do, so I have had bad consequences.”). Participants rated the items from 1 (strongly agree) to 5 (strongly disagree); higher scores indicated a greater tendency toward mobile phone addiction. Confirmatory factor analysis indicated that the model demonstrated a good fit to the data: χ^2^ (d*f* = 112) = 893.63; *p* < 0.05; comparative fit index (CFI) = 0.93; Tucker-Lewis index (TLI) = 0.92; root mean square error of approximation (RMSEA) = 0.07, 90% CI [0.06, 0.08]. The internal consistency reliability (α) was 0.86.

#### Parental rearing behavior

This study applied the revised Chinese version of the simplified parenting questionnaire compiled by Perris et al. (1980) and revised by Jiang et al. (2010) in China, using a four-point scale from 1 (never) to 4 (always). According to the principal component analysis (Markus et al., 2003; Jiang et al., 2010), parenting was divided into four dimensions: mother control (e.g., “Did it happen that your mother punished you more than you had deserved?”), mother care (e.g., “Did your mother expect you to become the best?”), father control (e.g., “Did it happen that your father punished you more than you had deserved?”), and father care (e.g., “Did your father expect you to become the best?”). The score of parental control was formed by averaging the scores of mother control and father control, and the score of parental care was formed by averaging the scores of mother care and father care. The higher the score, the higher the frequency of parental control and parental care. The Cronbach's alpha coefficient of the revised Chinese version of the simplified parenting questionnaire was 0.84 (Jiang et al., 2010). Confirmatory factor analysis indicated that parental control demonstrated a good fit to the data: χ^2^ (*df* = 240) = 1,491.66; *p* < 0.05; CFI = 0.92; TLI = 0.90; RMSEA = 0.06, 90% CI [0.05,0.07]. Meanwhile, parental care demonstrated a good fit to the data: χ^2^ (*df* = 103) = 857.86; *p* < 0.05; CFI = 0.93; TLI = 0.92; RMSEA = 0.07, 90% CI [0.06, 0.08]. The internal consistency reliability (α) of parental control and parental care in the present study was 0.79 and 0.75, respectively.

#### Self-control

The Chinese version of the Brief Self-Control Scale (BSCS) was used to assess self-control (Tangney et al., 2004; Tan and Guo, 2008). The BSCS contains 19 items with five dimensions: self-discipline (6 items, e.g., “I am good at resisting temptation”), inclination toward deliberate/non-impulsive action (4 items, e.g., “I never allow myself to lose control.”), an inclination toward healthy habits (3 items, e.g., “I am good at resisting temptation Healthy Habits”), inclination toward self-regulation in service of work or study (3 items, e.g., “I can work effectively toward long-term goals.”), and reliability (3 items, e.g., “I get carried away by my feelings.”). Participants rated the items from 1 (not at all) to 5 (very much). After scoring reverse items, higher scores indicated higher self-control. Confirmatory factor analysis indicated that BSCS has a good fit to the data: χ^2^ (d*f* = 94) = 451.26, *p* < 0.05; CFI = 0.94; TLI = 0.92; RMSEA = 0.05, 90% CI [0.04, 0.06]. The internal consistency reliability (α) in the present study was 0.80.

#### Future time perspective

The future time perspective questionnaire compiled by Song, 2004 was used to assess future time perspective. The scale contains five dimensions: behavioral commitment (e.g., “ I have a goal every day.”), future effectiveness (e.g., “I believe that I can build my beautiful future.”), far goal orientation (e.g., “I often envision goals to be achieved in five years.”), future intention (e.g., “I know there are many tasks to be completed in the future.”), and purpose awareness (e.g., “I have a vague idea of my future.”). Participants rated the items from 1 (strongly agree) to 5 (strongly disagree). After scoring reverse items, higher scores indicated a higher future time perspective. Confirmatory factor analysis indicated that the two-factor model demonstrated a good fit to the data: χ^2^ (d*f* = 66) = 638.27; *p* < 0.05; CFI = 0.95; TLI = 0.93; RMSEA = 0.08, 90% CI [0.07, 0.09]. The internal consistency reliability (α) in the present study was 0.76.

### Analysis process

The data was analyzed in the following steps. First, SPSS (v.22, IBM, Chicago, IL) was used to obtain the descriptive statistics of the data. Second, SEM using Mplus 7.4 was performed to test direct effects and mediation effects. Then, the latent moderated structural equations (LMS) method was used to specify the moderated mediation effects (Klein and Moosbrugger, 2000). In this procedure, a significant non-zero product term indicates the presence of an interaction. Moreover, because overall model fit indices, such as the chi-square statistic, CFI, and RMSEA, cannot be estimated using the LMS (Klein and Moosbrugger, 2000; Perren et al., 2013), the moderated mediation effect test was followed by the procedure described in previous studies (Perren et al., 2013), in which two models were tested to assess model fit. The first model was tested without the latent variable interaction (restricted model); the second model was tested with the latent variable interaction (full model). A likelihood ratio (LR) test was used to determine whether the inclusion of the interaction terms improved the model fit. Simulation studies demonstrated that the standard error estimates of the LMS remain relatively unbiased even when some variables are non-normal (Brandt et al., 2014). Full information maximum likelihood (FIML) estimation was used to treat missing data, which allowed for all sample participants to be retained in the study. The missing data of variables are presented in Table 1. The SES and gender of the college students were included as covariates. All the predictors were mean-centered to reduce multicollinearity. A significant interaction was further investigated using a simple slope technique. Moreover, the Johnson-Neyman technique (Bauer and Curran, 2005) was also used to plot a simple effect graph to better understand the moderating effects on the mobile phone addiction tendency.

**Table 1:** The missing data of variables.

**Variables**	**Missing data**
Mother control	9
Father control	11
Mother care	9
Father care	11
Self-control	11
Future time perspective	12
Mobile phone addiction	15

## Results

### Common method biases

To avoid common method biases, several strategies have been used, such as collecting anonymous responses and scoring some items in reverse. Furthermore, we applied Harman's single-factor test to evaluate the common method biases. The results showed that there were 15 factors with the original root greater than 1. The first factor could explain 21.26% of the cumulative variance, and the critical value was less than 40%. The result showed no severe common method biases in the present study (Zhou and Long, 2004).

### Description statistics

The means and SDs of all variables and the correlations between these variables are presented in Table 2.

**Table 2:** Descriptive statistics and correlations among variables.

	**1**	**2**	**3**	**4**	**5**	**6**	**7**	**8**	**9**
1 Sex	1								
2 SES	0.07	1							
3 Mother control	0.15**	0.05	1						
4 Mother care	−0.10*	0.28**	−0.24***	1					
5 Father control	0.20**	0.08*	0.67***	−0.19**	1				
6 Father care	−0.07*	0.20**	−0.13**	0.60**	−0.14**	1			
7 Self-control	−0.01	0.05	−0.28**	0.25**	−0.24**	0.22**	1		
8 Future time perspective	0.02	0.13**	−0.11**	0.43**	−0.07**	0.36**	0.36**	1	
9 Mobile phone addiction tendency	0.04	0.02	0.40**	−0.17**	0.35**	−0.16**	−0.51**	−0.19**	1
*M*	-	-	22.98	21.93	21.64	20.19	60.72	39.95	41.56
SD	-	-	6.66	5.33	6.29	5.01	10.02	8.33	11.63

*SES*, Socioeconomic status, ^*^p < 0.05, ^**^p < 0.01, ^***^p < 0.001.

### Testing direct relations

The results showed that mother control was positively associated with mobile phone addiction tendency (β = 0.40, *p* < 0.01) and that father control was positively associated with mobile phone addiction tendency (β = 0.35, *p* < 0.01; 95% CI [0.41, 0.56]). Whereas, the results showed that mother care was negatively associated with mobile phone addiction tendency (β = −0.17, *p* < 0.01) and that father care was negatively associated with mobile phone addiction tendency (β = −0.16, *p* < 0.01). Considering that the mother and father have a similar effect on adolescents' mobile phone addiction tendencies, we combined the mother control and the father control to latent variable parental control and mother care and father care to latent variable parental care.

The results showed that parental control (combined with mother control and father control) was positively associated with mobile phone addiction tendency (β = 0.48, *p* < 0.001; 95% CI [0.41, 0.56]) and that parental care (combined with mother care and father care) was negatively associated with mobile phone addiction tendency (β = −0.09, *p* = 0.02; 95% CI [−0.17, −0.01]).

### Testing the mediation model

The overall fit of the mediation model was adequate (χ^2^ = 616.10, d*f* = 70, *p* < 0.001; TLI = 0.90, CFI = 0.92, RMSEA = 0.07, and SRMR = 0.05). The mediation analysis showed that parental control was indirectly positively related to mobile phone addiction tendency through self-control after controlling for gender and SES of adolescents (β_ind_ = 0.15, *p* < 0.05; 95% CI [0.11, 0.19]). Meanwhile, parental care was indirectly negatively related to mobile phone addiction tendency through self-control after controlling for gender and SES of adolescents (β_ind_ = −0.11, *p* < 0.05; 95% CI [−0.17, −0.06], see Table 3). The 95% CI did not show zero, meaning that self-control partially mediates the association between parental care and mobile phone addiction tendency. The results showed that **Hypothesis 1** was supported.

**Table 3:** Summary for the mediation model.

	**Self-control**			**Mobile phone addiction tendency**		
**Variable**	**β**	**SE**	**95%CI**	**β**	**SE**	**95%CI**
Parental control	−0.28***	0.04	[−0.36, −0. 18]	0.33***	0.04	[0.26, 0.40]
Parental care	0.30***	0.05	[0.20, 0.40]	0.02	0.04	[−0.06, 0.11]
Family SES			-	−0.01	0.03	[−0.12, 0.21]
Adolescents sex			-	−0.01	0.03	[−0.01, 0.30]
Self-control			-	−0.49***	0.04	[−0.58, −0.42]
Future time perspective			-	−0.01	0.03	[−0.09, 0.06]
Self-control × Future time perspective			-	−0.05*	0.02	[−0.10, −0.007]

^*^p < 0.05, ^***^ *p* < 0.001.

### Testing the moderated mediation model

**Figure 2:**
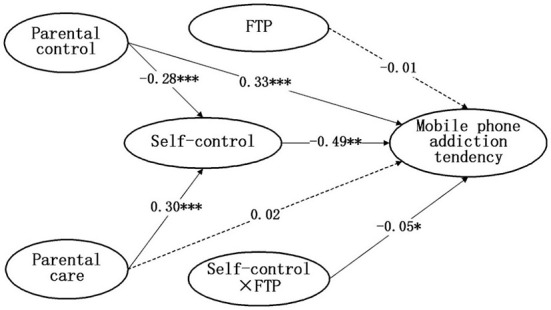
The moderated mediation analysis of self-control on the relationship between parental control and parental care and adolescents' mobile phone addiction tendencies conditioned by FTP.

**Figure 3:**
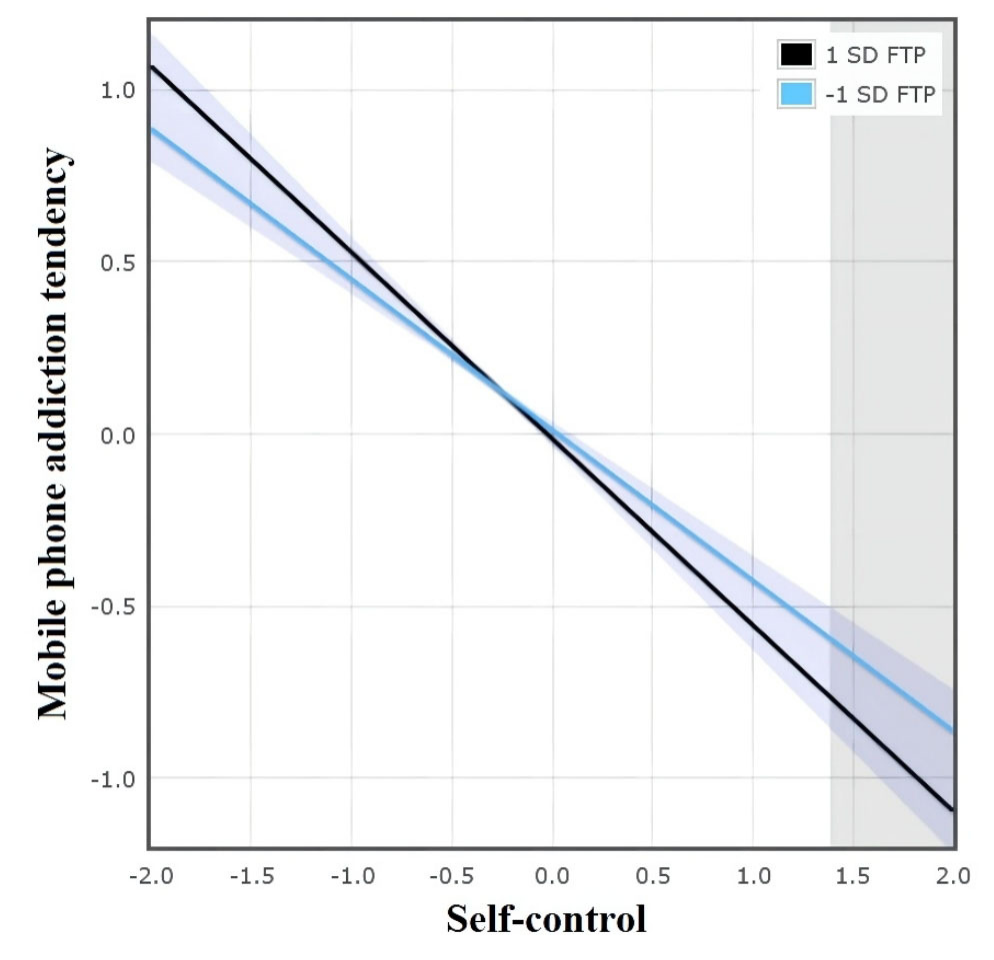
The simple slope of self on the mobile phone addiction tendency at different levels of FTP.

**Figure 4:**
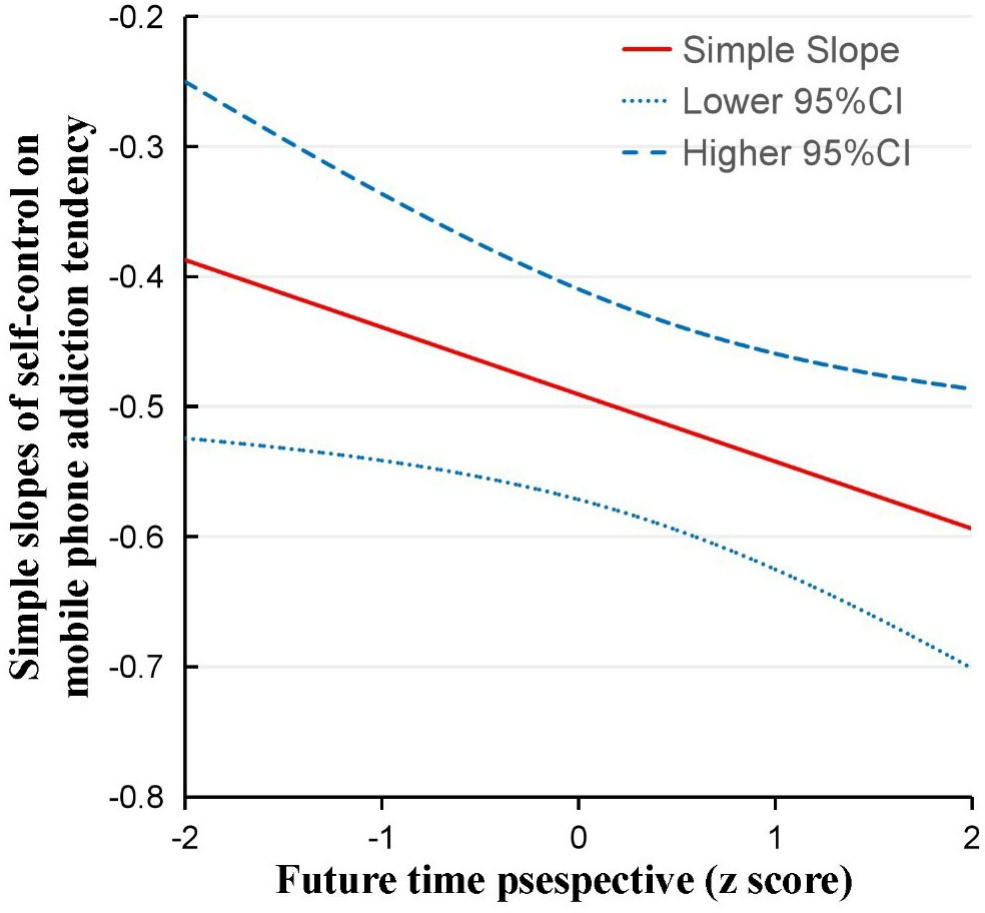
The Johnson-Neyman technique showed the simple slope of self-control on the mobile phone addiction tendency. Dotted lines represent upper and lower 95% CIs of the simple slope. A high future time perspective combined with high self-control predicted a low level of mobile phone addiction tendency.

The latent moderated structural equations model results showed that the moderating effects of future time perspective between self-control and mobile phone addiction tendency were significant (β= −0.05, *p* = 0.02). Two models of each condition were estimated to assess the interaction effect model fit. The first model had an adequate fit: χ^2^ (d*f* = 125) = 1,059.54, RMSEA = 0.07, 90% CI [0.07, 0.08], CFI = 0.92, TLI = 0.90, and SRMR = 0.07. Then, LR tests were used to compare whether the inclusion of the interaction terms improved the model fit. The model without latent interaction effects had a LogL (Restricted) = −27,254.08, and the model with the interaction effect had a LogL (Full) = −27,250.26, LR (d*f* = 1) = 3.46, and *p* = 0.06. This result suggested that including the interaction effects under both conditions improved the overall model fit. It showed that the future time perspective moderated the indirect pathway between parental control/parental care and self-control and adolescents' mobile phone addiction tendencies, and thus, Hypothesis 2 was supported. Specifically, a high future time perspective combined with high self-control predicted a low level of mobile phone addiction tendency. In contrast, low self-control was associated with a high tendency toward mobile phone addiction, irrespective of their future time perspective. The moderating effect of the future time perspective is presented in Table 4 and Figures 2–4.

**Table 4:** Summary for the moderated mediation effect.

	**Parental control → Self-control → Mobile phone addiction tendency**			**Parental care → Self-control → Mobile phone addiction tendency**		
	**Indirect**	**SE**	**95%CI**	**Indirect**	**SE**	**95%CI**
**Future time perspective**						
−1 SD	0.12***	0.02	[0.08, 0.17]	−0.12***	0.02	[−0.19, −0.08]
0 SD	0.14***	0.02	[0.09, 0.19]	−0.15***	0.03	[−0.21, −0.09]
+1 *S*D	0.15***	0.03	[0.10, 0.20]	−0.16***	0.03	[−0.23, −0.10]

***p < 0.001.

## Discussion

Overall, the present study found that self-control partially mediated the relationship between parenting (including parental control and parental care) and adolescents' mobile phone addiction tendencies. Similarly, the future time perspective moderated the mediation pathway between self-control and adolescents' mobile phone addiction tendencies. These findings extend the previous literature by demonstrating that combined family environmental and individual factors contribute to the likelihood of adolescents' mobile phone addiction tendencies (Liu et al., 2020; Sun et al., 2020).

Consistent with previous studies (Mousavi et al., 2015; Lee et al., 2016; Lian et al., 2016), the present study found that parental control was positively related to adolescents' mobile phone addiction tendencies, while parental care was negatively related to adolescents' mobile phone addiction tendencies. The present study found that mothers and fathers have a similar impact on adolescents. Therefore, both mother care and father care characterized by affectionate warmth, acceptance, identification, and support may facilitate the adaptive development of adolescents. Both mother and father control characterized by control, rejection, and domination may hinder the development of adolescents. Together with previous studies (Li et al., 2014; Mousavi et al., 2015; Lee et al., 2016; Lian et al., 2016), the findings suggested that parental rearing behavior as a crucial family environment factor could have a profound impact on behavioral or technological addiction of adolescents, including mobile phone addiction tendency.

The present study found that self-control mediated the relationship between parental rearing behavior (including parental control and parental care) and Chinese adolescents' mobile phone addiction tendencies. Self-control is regarded as an important aspect of cognitive development through adolescence (Diamond, 2013) and is critical to successful functioning in almost every aspect of the development of adolescents (Gottfredson and Hirschi, 1990; Muraven and Baumeister, 2000). Thus, self-control could be an important bridge between parental rearing behavior and adolescents' mobile phone addiction tendencies. On the one hand, self-control in adolescents could be obstructed by both mothers and fathers being excessively interfering or controlling (Barber, 2002; Crosswhite and Kerpelman, 2012; Li et al., 2013). Consequently, adolescents with poor self-control may become prone to the overuse of mobile phones and cause mobile phone addiction tendency when facing stimuli and temptation from the visual world (Sok, 2019). On the other hand, parental care as supportive parenting could enhance the self-control ability in adolescents (Li et al., 2019), reducing the likelihood of mobile phone addiction. Self-control is thus an important mediator linking parenting and Chinese adolescents' mobile phone addiction tendencies.

Furthermore, the present study found that the future time perspective moderated the indirect pathway between parental control/parental care and self-control to adolescents' mobile phone addiction tendencies. Specifically, a high future time perspective combined with high self-control predicted a low level of mobile phone addiction tendency. As an influential motivation factor, a high future time perspective not only lets adolescents better plan and monitor current behavior but also could compensate for the exhaustion of self-control resources (Dreves and Blackhart, 2019), which reduces the addiction risk of using mobile phones. In contrast, low self-control was associated with a high tendency toward mobile phone addiction, regardless of their future time perspective. This finding provided empirical support for the protective-protective model, which proposed that the two individual protective factors could act in combination, that is, one protective factor could enhance the role of another protective factor (Cohen, 2003). Specifically, a high level of future time perspective could strengthen the protective effect of a high level of self-control on mobile phone addiction tendencies.

Overall, by examining the mediating role of self-control and the moderating role of future time perspective, the present study extended several theories, such as the self-control theory (Gottfredson and Hirschi, 1990; Hirschi and Gottfredson, 1993), the dual-motive model of self-control (Fujita, 2011), and the strength model of self-control (Baumeister et al., 2007), and the research field of mobile phone addiction tendency. This study further revealed that an upbeat parental rearing behavior, such as parental care, and a negative parental rearing behavior, such as parental control, could influence self-control in adolescents, which may, in turn, affect their mobile phone addiction tendency. Notably, a high future time perspective plays a strengthening role rather than a buffering role in the association between self-control and mobile phone addiction tendencies. The present study enriched our knowledge of how and when parental rearing behavior contributes to adolescents' mobile phone addiction tendencies.

In line with the component model of behavioral addiction theory (Griffiths, 2005), the present study further revealed that the formation of mobile phone addiction tendency lies in the complex interaction between family environmental factors and individual factors. A higher level of future time perspective moderated the relationship between parental control/parental care, self-control, and mobile phone addiction. These findings supported that the link between parental rearing behavior and adolescents' mobile phone addiction tendencies might depend on other individual psychological factors. In addition, self-control was partially mediated by the relationship between parental control and adolescents' mobile phone addiction tendencies. As previous studies suggested (Billieux, 2012), the etiology of mobile phone addiction tendency is complex and may involve various potential psychological mechanisms. The findings suggested that there are other potential psychological mechanisms between parental control and adolescents' mobile phone addiction tendencies, and future studies should be further explored.

There are several limitations to the present study. The first limitation is that, although the moderated mediation model examined in the present study is based on theoretical and empirical research, the cross-sectional design could not determine the causal relationships between variables. Longitudinal studies should clarify the causal relationships investigated in the present study. The second limitation is that the present study collected self-control from self–reported questionnaires; future studies can use experiments to assess self-control simultaneously.

The present study is the first attempt to examine the mediating role of self-control and the moderating role of the future time perspective in the link between positive and negative parental rearing behavior and mobile phone addiction tendencies of Chinese adolescents. It could advance our understanding of how and when parental rearing behavior is associated with mobile phone addiction tendencies among adolescents. Furthermore, the present study could enrich relevant studies on the association between environmental factors and adolescents' mobile phone addiction by comprehensively examining the mediating role of self-control and the moderating role of future time perspective in such association.

The present study could also provide guidance for preventing and intervening in adolescents' mobile phone addiction tendencies. First, interventions that aim to alleviate mobile phone addiction in adolescents should be started early by enhancing positive parental rearing behavior, such as parental care, and reducing negative parental rearing behavior, such as parental control, which has a persistent impact on children. Second, the finding demonstrated that self-control is a critical mediator linking parenting with adolescents' mobile phone addiction tendencies. This finding suggested that self-control may be a fruitful intervention target for reducing adolescents' mobile phone addiction tendencies. Previous studies exhibited that there are two main strategies to foster self-control. One is a cognitive approach and the other is through behavioral and socioemotional training (Beames et al., 2018; Pesce et al., 2020). Practitioners could conduct skills training on self-control to help adolescents improve their effective behavior and emotion control strategies. Third, the finding revealed that the future time perspective moderated the relationship between self-control and adolescents' mobile phone addiction tendencies. The present study suggested that effective intervention programs could enhance adolescents' future time perspective to facilitate self-control and reduce the risk of mobile phone addiction. Overall, adolescents who exhibited poor future time perspectives and experienced a high level of parental control and poor self-control may benefit most from future prevention programs to reduce their mobile phone addiction.

The present study contributed to the understanding of the potential psychological mechanism underlying the link between parenting and s' mobile phone addiction tendencies of Chinese adolescents. Mediation analysis indicated that self-control may be an explanatory factor for why a negative parental rearing behavior impacts adolescents, and a positive parental rearing behavior could also prevent mobile phone addiction tendency among adolescents. In addition, moderated mediation analysis revealed that future time perspective could moderate the indirect pathway between parental rearing behavior, self-control, and mobile phone addiction tendency among Chinese adolescents.

## Data availability statement

The original contributions presented in the study are included in the article/supplementary materials, further inquiries can be directed to the corresponding authors.

## Ethics statement

The studies involving human participants were reviewed and approved by the Research Ethics Committee of Xi'an University approved this study. Written informed consent to participate in this study was provided by the participants' legal guardian/next of kin.

## Author contributions

All authors listed have made a substantial, direct, and intellectual contribution to the work and approved it for publication.

## Funding

This research was supported by the MOE (Ministry of Education in China) Project of Humanities and Social Sciences (22XJC190002), the Natural Science Basic Research Plan in Shaanxi Province of China (2022JQ-193), and the Xi'an Social Science Planning Fund (2021ZDZT16).

## Conflict of interest

The authors declare that the research was conducted in the absence of any commercial or financial relationships that could be construed as a potential conflict of interest.

## Publisher's note

All claims expressed in this article are solely those of the authors and do not necessarily represent those of their affiliated organizations, or those of the publisher, the editors and the reviewers. Any product that may be evaluated in this article, or claim that may be made by its manufacturer, is not guaranteed or endorsed by the publisher.
